# KIT (CD117) Positive Huge Primary Malignant Extra Gastrointestinal Stromal Tumors (EGISTs) Arising From Jejunal Mesentery: A Rare Case Report

**DOI:** 10.7759/cureus.33168

**Published:** 2022-12-31

**Authors:** Anita B Sajjanar, Nilesh T Katole, Sunita J Vagha

**Affiliations:** 1 Pathology, Datta Meghe Medical College, Datta Meghe Institute of Medical Sciences, Nagpur, IND; 2 Pharmacology and Therapeutics, Datta Meghe Medical College, Datta Meghe Institute of Medical Sciences, Nagpur, IND; 3 Pathology, Jawaharlal Nehru Medical College, Datta Meghe Institute of Medical Sciences, Wardha, IND

**Keywords:** immunohistochemistry, histopathology, mdct scan, mesentery, jejunum, extra gastrointestinal stromal tumor, malignant

## Abstract

Tumors arising outside gastrointestinal systems are known as extra gastrointestinal stromal tumors (EGISTs). Outside gastrointestinal sites include the mesentery, omentum, peritoneum, pancreas, and liver. Our case highlights a rare occurrence of an EGIST in jejunal mesentery in a 45-year-old male with an asymptomatic large abdominal growth and weight loss. A contrast-enhanced multi-dimensional computed tomography scan showed a large heterogeneous mass in the left hypochondrium. lumbar, and paraumbilical regions. Later, the patient underwent surgical resection of the tumor along with the involved jejunal segment and small tumor masses in the mesentery. Histopathological examination reported a malignant EGIST of mesentery and invasion into the jejunum, further confirmed by immunohistochemistry (IHC) markers like CD117 and smooth muscle actin with a high proliferative index (Ki67). One should be aware that these are different from other malignancies arising from the mesentery. Their cell of origin is different and needs a specific type of treatment. The clinical history, radiological findings, histopathology, and IHC help in diagnosing especially when they are arising from unusual areas like jejunal mesentery. Surgical intervention and chemotherapy are mainstay treatments.

## Introduction

Mazur and Clark used the term gastrointestinal stromal tumor (GIST) for the first time in 1983 [1]. These are mesenchymal-origin neoplasms primarily occurring in the alimentary system. The commonest site is the stomach (60-70%), followed by the small intestine (20-25%), colon and rectum (5%), esophagus (<5%), and liver [2]. The GIST that originates outside the alimentary system is known as an extra gastrointestinal stromal tumor (EGIST) [3].

CD117 (c-kit protein) is expressed by interstitial cells of Cajal that are thought to be the origin of GISTs, similar to EGISTs [4]. Although GISTs may occur at any age, most commonly they are encountered in and around the sixth to the seventh decade of life. The clinical manifestations include Melena, abdominal pain, and anemia.

GISTs have variable behavior ranging from benign to malignant potential [5]. Malignant GISTs have the potential to metastasize unusual sites and are found to be locally infiltrative [6]. The prognostic indicators include size, mitotic index, stage, proliferative index, and recurrence after treatment [7].

GISTs are slow-growing tumors, which usually present nonspecific symptoms like slow growing abdominal mass, abdominal distension, pain, and weight loss, making them very difficult to diagnose clinically. The literature review revealed very few cases of EGISTs from jejunal mesentery. This case highlights a rare occurrence of a huge EGIST in jejunal mesentery which presents asymptomatic, painless, large abdominal growth. It was diagnosed by histopathology and confirmed by immunohistochemistry (IHC) which helps in further management.

## Case presentation

A 45-year-old male patient came with a mass in the abdomen and weight loss for over two months. Physical examination findings indicated a soft, painless bulge in the left hypochondrium and lumbar regions. A multidimensional computed tomography showed large, well-defined, lobulated, mixed density, heterogeneously enhancing mass occupying the left hypochondrium, lumbar, and paraumbilical regions and crossing the midline to extend into the right lumbar region.

**Figure 1:**
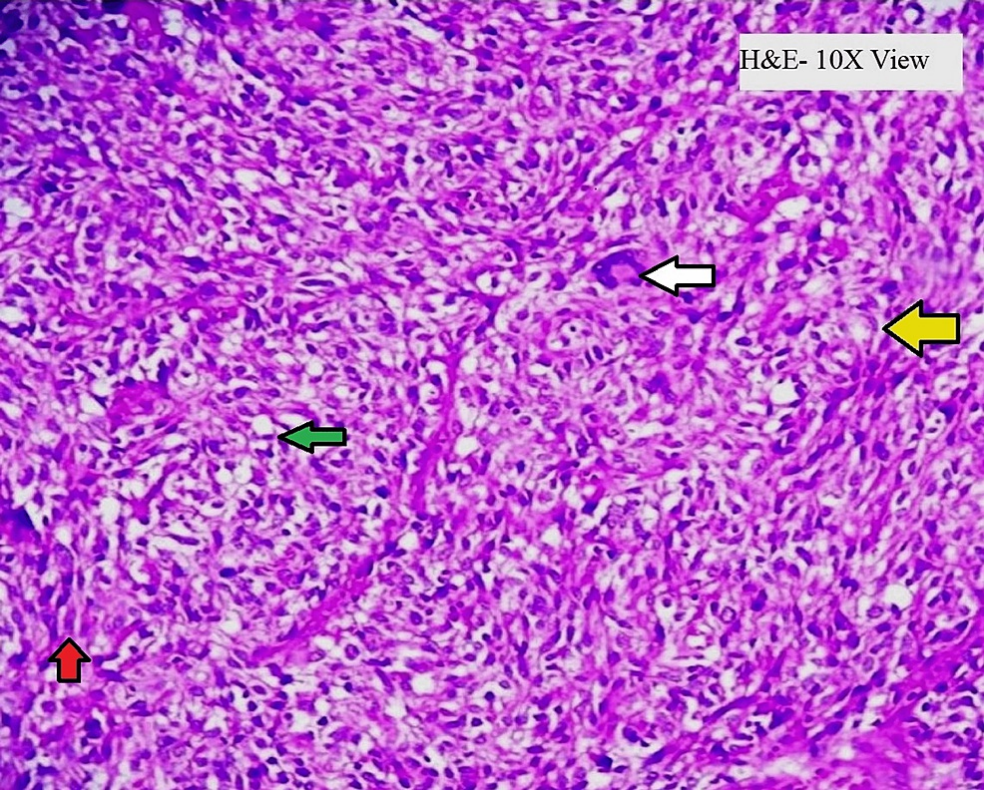
Microscopic histopathology shows tumor cells arranged in a whorl pattern (yellow arrow), composed of spindle cells (red arrow) and epithelioid cells (green arrow). Multinucleated tumor giant cells (white arrow) are also seen. Hematoxylin- and eosin-staining and 10x magnification are done.

**Figure 2:**
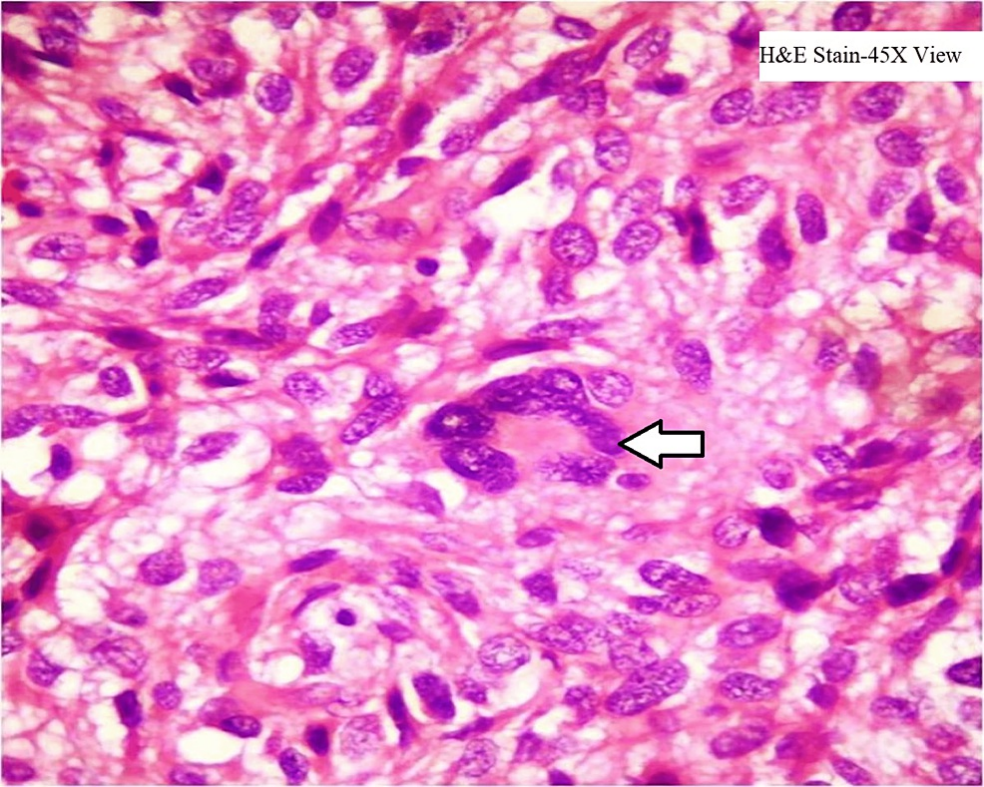
Microscopic histopathology shows tumor cells with some multinucleate tumor giant cells (5-6 nuclei). Hematoxylin- and eosin-staining and 45x magnification are done.

The microscopic examination showed malignant cells in the form of diffuse sheets and whorls (Figure 1) which are round to polygonal having pleomorphic, hyperchromatic nuclei and 1 to 2 inconspicuous nucleoli and clear cytoplasm and 5-6 mitotic figures per 50 HPF along with few multinucleate tumor giant cells (Figure 2). Areas of hemorrhage and necrosis are seen along with proliferating blood vessels. Sections from the jejunum showed normal mucosa and tumor invasion in lamina propria, submucosa, muscularis propria, and serosa.

**Figure 3:**
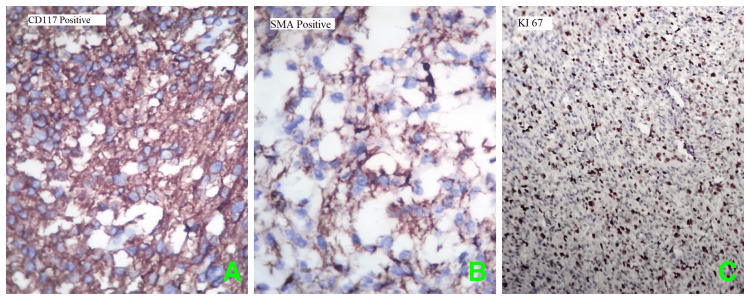
Immunohistochemical staining showed diffuse strong cytoplasmic positivity for A) CD117 positive, B) SMA positive, and C) high Ki 67 proliferation index (40% -50%) in the nucleus (40x magnification). SMA: Smooth muscle actin

Further examination using IHC markers showed marked expression for CD117 (C-KIT) (Figure 3A) and smooth muscle actin (SMA) (Figure 3B) while negative for S100. The Ki 67 proliferative index shows 40%- 50% tumor cells (Figure 3C), thus confirming the diagnosis as EGISTs.

## Discussion

GISTs are rare neoplasms that represent up to one percent of all gastrointestinal tumors [8]. They rarely (5%) originate outside the GI tract as primary tumors described as extra-GISTSs (EGISTs) which are histologically and immunophenotypically similar to GI stromal tumors [9]. They are thought to originate from interstitial cells of Cajal (pacemaker for autonomous gastrointestinal motility) which expresses CD117 (KIT). CD117 (KIT) is a tyrosine kinase encoded by protooncogene c-KIT. Thus, these are double-positive for KIT and CD34 markers. They occur in sporadic as well as familial forms. In the sporadic form, 90% are from c-kit somatic mutation (gain of function) while in the familial form germline gain-of-function mutations of the c-kit gene are seen [10]. In our case, it was sporadic in origin.

Common sites of EGIST include omentum, mesentery, retroperitoneum, liver, pancreas, and prostate [11,12]. Only 114 cases of GISTs with mesenteric origin have been recorded in the literature to date [13], and among these, only a few are about jejunum sac EGISTs [14]. Mesentery-related EGISTs behave more aggressively than stomach or small-bowel-related GISTs. Most of the mesenteric GISTs are larger than 10 cm in size at initial presentation and have a higher mitotic index (>5 mitotic figures/50 HPF) [13].

The diagnosis of mesenteric GISTs is challenging since these tumors lack distinctive clinical or radiological characteristics. They are usually first identified postoperatively, while gastrointestinal bleeding from mucosal ulcerations or fistula is a common consequence of GISTs. The EGIST typically has no symptoms unless nearby tissues are squeezed [3]. According to a review done by Iqbal et al., abdominal pain is the most common symptom of EGISTs, followed by a palpable abdominal mass and occasionally anemia or lower limb edema [11]. Our patient had abdominal distention and weight loss as major presenting complaints, but there was no history of pain in the abdomen as seen in many previous cases. The EGIST is usually present in large sizes; the mean size is 10 cm and may reach up to 32 cm in size [3]. In our case, the tumor size was huge, measuring 25x22x18cms and weighing about 3.3kgs which as per our knowledge could be one of the heaviest EGISTs reported to date.

Contrast computed tomography shows predominantly large solid mass displacing the nearby structures, with intense peripheral enhancement, of undetermined origin [15]. Because of the large size and the enhancement, they can mimic colonic neoplasms as seen in our case.

Histopathology and IHC are the gold standards for the diagnosis of EGISTs. Histopathological findings mostly demonstrate three variants: epithelioid, spindle cell, or mixed type. An epithelioid variant is formed in diffuse sheets or nests. They are rounded with vesicular nuclei and clear to eosinophilic cytoplasm. Spindle cell variants are in intersected fascicles and whorls. The individual cells have ovoid nuclei, fine nuclear chromatin, inconspicuous nucleoli, and eosinophilic cytoplasm. The stoma may exhibit areas of myxoid change, necrosis, and hemorrhage. In a mixed type, a mixture of both epithelioid and spindle cells is seen as seen in our case [16].

In an IHC study, CD117 (c-KIT) staining positivity is necessary for GIST diagnosis. Other markers include BCL-2 (80%), CD34 (70%), SMA (30%), desmin (5%), and DOG1 [11,17,18]. The DOG1 (Discovered on GIST), the latest monoclonal antibody, is highly expressed in both CD117 positive and negative tumors, which have a mutation in platelet-derived growth factor receptor-alpha (PDGFRA). Thus, the newer marker DOG1 improves diagnostic accuracy, especially in KIT-negative tumors [19]. 

Differential diagnoses include abdominal tumors like colonic tumors, lymphoma, liposarcoma, histiocytoma, leiomyosarcoma, fibrosarcoma, etc. Mesenteric fibromatosis is closer differential to EGISTs considering a similar immunohistochemistry pattern except for β catenin expression which is absent in EGISTs. Unlike EGISTs, mesenteric fibromatosis is much more invasive with a high rate of recurrence [20].

Although cellularity, tumor mass, mitotic activity (>2/50 HPF), and necrosis have been suggested as potential prognostic factors, it is evident that additional research with a larger sample size is required to draw reliable conclusions on prognostic factors [11,16]. Our patient was categorized as having high-risk EGISTs because of the size of the tumor and the patient's high mitotic index (5-6/50 HPF), high cellularity, and areas of necrosis.

There are currently no established management guidelines for EGISTs. As almost all patients of EGISTs are considered high risk, surgical debulking of the tumor with an infiltrated structure and macroscopically negative margins followed by adjuvant imatinib is a mainstay of EGIST management [8,13]. In our case, en-block resection of the tumor with a jejunal section was done. Postoperatively, the patient recovered and was discharged with imatinib therapy.

## Conclusions

Therefore, one should keep EGISTs in mind while handling a large painless abdominal mass. They can be easily missed if relied on clinical and radiological investigations. A low threshold of suspicion is necessary in order to diagnose this aggressive entity. IHC is the gold standard for the diagnosis of EGISTs. Surgery is the mainstay of treatment, and the role of imatinib is still unclear. More studies are required to fully understand its behavior and formulate treatment guidelines, and hence more and more case reporting is necessary. Early diagnosis and surgical debulking of tumor mass are crucial for the better survival of patients.
